# Microenvironmental acidosis in carcinogenesis and metastases: new strategies in prevention and therapy

**DOI:** 10.1007/s10555-014-9531-3

**Published:** 2014-11-07

**Authors:** Stefano Fais, Giulietta Venturi, Bob Gatenby

**Affiliations:** 1 Department of Therapeutic Research and Medicines Evaluation, Unit of Antitumor Drugs, Istituto Superiore di Sanità, Viale Regina Elena 299, Rome, Italy; 2 Radiology Department, Cancer Biology and Evolution Program Moffitt Cancer Center, 12902 Magnolia Drive, Tampa, FL 33612 USA; 3 Department of Drug Research and Medicines Evaluation, Istituto Superiore di Sanità (National Institute of Health), Viale Regina Elena 299, 00161 Rome, Italy

**Keywords:** Acidity, Cancer, Microenvironment, pH gradient, Proton export mechanisms, Proton pump inhibitors

## Abstract

Much effort is currently devoted to developing patient-specific cancer therapy based on molecular characterization of tumors. In particular, this approach seeks to identify driver mutations that can be blocked through small molecular inhibitors. However, this approach is limited by extensive intratumoral genetic heterogeneity, and, not surprisingly, even dramatic initial responses are typically of limited duration as resistant tumor clones rapidly emerge and proliferate. We propose an alternative approach based on observations that while tumor evolution produces genetic divergence, it is also associated with striking phenotypic convergence that loosely correspond to the well-known cancer “hallmarks”. These convergent properties can be described as driver phenotypes and may be more consistently and robustly expressed than genetic targets. To this purpose, it is necessary to identify strategies that are critical for cancer progression and metastases, and it is likely that these driver phenotypes will be closely related to cancer “hallmarks”. It appears that an antiacidic approach, by targetting a driver phenotype in tumors, may be thought as a future strategy against tumors in either preventing the occurrence of cancer or treating tumor patients with multiple aims, including the improvement of efficacy of existing therapies, possibly reducing their systemic side effects, and controlling tumor growth, progression, and metastasis. This may be achieved with existing molecules such as proton pump inhibitors (PPIs) and buffers such as sodium bicarbonate, citrate, or TRIS.

## Introduction

### The physical microenvironment in tumors

**Fig. 1:**
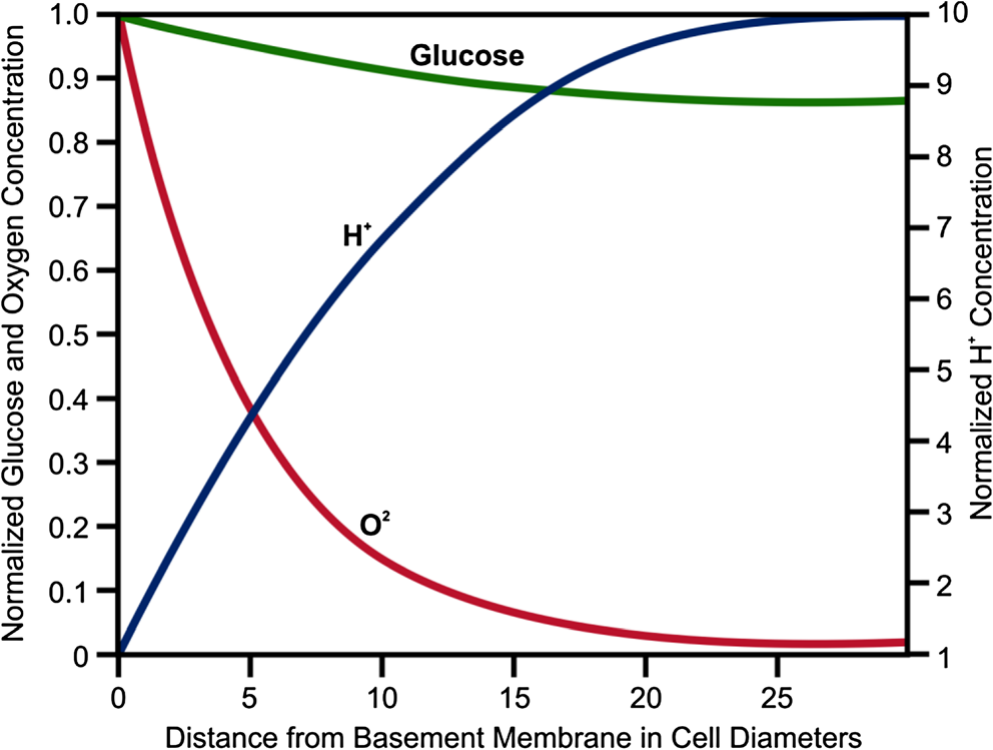
Spatial variations in glucose, oxygen, and H^+^ concentrations around a single intratumoral blood vessel

**Fig. 2:**
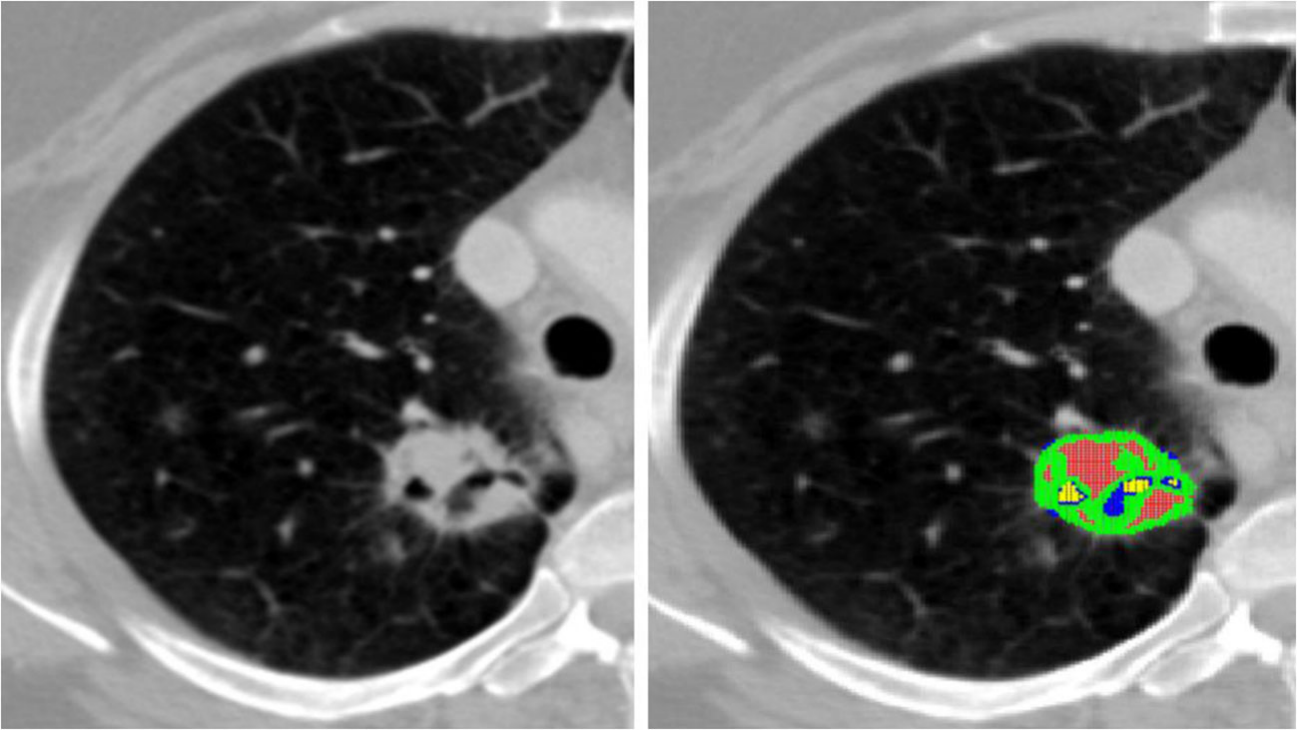
Computerized tomography scan from a lung cancer demonstrates intratumoral regions of necrosis (*left panel*). Image analysis (*right panel*) demonstrates corresponding variations in blood flow

All phases of the development and growth of tumors and their responses to therapies are critically influenced by the tumor physical microenvironment. Here, physical microenvironment refers to key substrate and metabolites (oxygen, glucose, and pH) as well as growth and regulatory factors which are typically transported to and from tissue primarily by the vascular system. The structure and function of the vasculature, therefore, strongly influence the physical microenvironment, and in cancers, there is marked spatial and temporal variation in blood flow [1]. In part, this is due to failure of the blood vessel formation (angiogenesis), and in part, it reflects chaotic blood flow due to failure of maturation in intratumoral blood vessels. In turn, this creates regional and temporal variations in environmental conditions with complex gradients of glucose, oxygen, H^+^, and other substrates and metabolites (Fig. 1). Tumor cell density is typically dependent on environmental conditions so that regions of almost total cell death (necrosis) are often visualized in tumors (Fig. 2).

Thus, tumor cells must adapt to a wide range of environments within tumors, and this is undoubtedly an important factor in the observed intratumoral molecular heterogeneity. However, cancer cells also play an active role in determining their environment, an evolutionary strategy termed “niche engineering” [2] (beaver dams being an obvious analogy in nature). Tumor cells often release increased levels of growth factors, which diffuse through the extracellular environment and cause characteristic changes in vascular growth.

Of importance here, cancer cells also commonly alter their environments through the use of anaerobic glucose metabolism [3] (i.e., glucose metabolism to lactic acid) even in the presence of normal oxygen concentrations. This has two specific consequences: (1) increased glucose flux to compensate for decreased efficiency in converting glucose to ATP; and (2) increased production of H^+^ ions, which must be extruded into the environment. As a result, cancers often maintain an acidic microenvironment even when vascular density and flow is relatively normal.

It is clear that cancers must be viewed not as a mass of cancer cells but as a complex society containing interacting populations of cancer and normal cells. Multiple studies have now demonstrated that improved understanding of these interactions can improve strategies for cancer prevention and treatment [4–7].

Although many examples of such interactions can be cited, here we focus on the role of extracellular pH as a mechanism by which the environment affects the cancer cells and vice versa. We particularly focus on potential therapeutic strategies that perturb these dynamics and alter tumor development and growth.

In this review, we will emphasize two major issues: (1) the role of tumor associated microenvironmental acidosis in governing tumor growth, invasion, and metastases; and (2) the role of acidosis in altering tumor response to therapy and potential treatment strategies targeting intratumoral acidosis.

### Aerobic glycolysis (the Warburg effect)

It is impossible to discuss the role of pH in cancer without first introducing Warburg’s nearly century old observations [3]. Briefly, mammalian cells can efficiently generate energy from glucose using oxygen to form CO_2_ and H_2_O, generating about 36 moles of ATP/mole of glucose. The alternative glycolytic metabolic pathway does not require oxygen so that each glucose molecule is converted to two molecules of lactate generating only two molecules of ATP. In 1867, Pasteur demonstrated that yeast decreases ethanol production following “aeration” of the culture media. This observation led to an enduring paradigm that in the absence of pathology, cells optimize the efficiency of ATP production within environmental constraints. Thus, high efficiency oxidative phosphorylation (up to 36 ATP/ glucose) is generally assumed to be the default source of ATP under physiological conditions, whereas the Embden-Meyerhoff fermentative (glycolytic) pathway, glycolysis (two ATP/glucose), is the “emergency backup” to be used only when oxygen is deficient. Warburg first noted that transformed cells are an exception to these principles. That is, cancer cells frequently exhibit high rates of lactate production even in the presence of oxygen (aerobic glycolysis).

The Warburg effect was originally ascribed to a failure of oxidative metabolism [8], but mitochondrial dysfunction is observed in only a small subset of cancers [9, 10]. Alternatively, it has been suggested that the Warburg effect, through its production of lactate, provides necessary carbon substrate for biosynthesis of macromolecules [11, 12]. However, experimental observations have demonstrated that only a very small percentage of lactate molecules produced by aerobic glycolysis (<7 %) is retained in the cancer cell and glutamine serves as the major carbon source [13]. Furthermore, aerobic glycolysis is commonly observed in normal, proliferating, and non-proliferating cells [9, 14–16]. Thus, although Warburg first observed of aerobic glycolysis over 70 years ago, its biological basis in cancer and normal cells remains unclear [15].

For decades, the Warburg effect, although well recognized, was largely relegated to a laboratory curiosity. However, interest in aerobic glycolysis has significantly expanded with the advent of widespread application of FdG-PET imaging. It is now clear that the vast majority of clinical cancers exhibit increased glucose uptake and, as a consequence of increased aerobic glycolysis, are significantly more acidic than normal tissue [15]. Many investigations of the molecular mechanism of the Warburg effect have provided insights into how aerobic glycolysis emerges in cancer cell. Less clear is the question of why it develops. That is, in the conventional model of carcinogenesis as an evolutionary process, it is initially difficult to understand the Darwinian dynamics leading to consistent selection for aerobic glycolysis which is less energetically efficient than oxidative phosphorylation and produces large amounts of acid. The latter requires energy to be exported from the cell and results in a potentially toxic acidic microenvironment since most mammalian cells cannot survive prolonged exposure to an extracellular pH lower than 7.2.

**Fig. 3:**
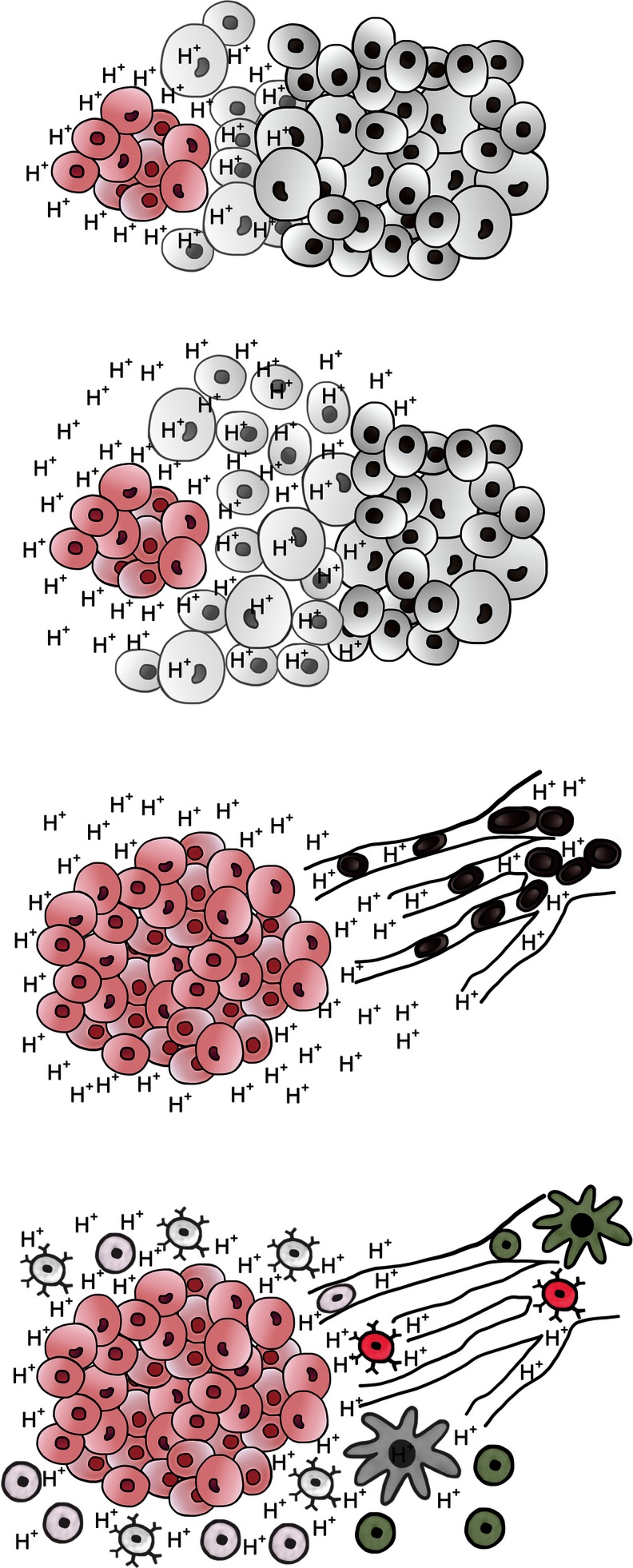
Acid-mediated tumor invasion. Increased glycolysis by cancer cells produces and acidic microenvironment. H^+^ flows along concentration gradients into adjacent normal tissue causing **a** normal cell death (*top*), **b** extracellular matrix degradation, and **c** angiogenesis. All of these responses promote tumor growth and invasion

We and others [4, 16–18] have addressed this evolutionary conundrum with the fundamental assumption that due to the Darwinian dynamics that govern somatic evolution, every common phenotype observed in cancer populations must confer an adaptive advantage. With insights provided by mathematical models, we have proposed that the Warburg effect increases the fitness of cancer cells through a number of mechanisms. One such mechanism involves potential advantages of acid production. In general, we propose that aerobic glycolysis represents an evolutionary strategy described as “niche engineering” in which a population generates environmental properties that decrease the fitness of its competitors. That is, the cancer cells having evolved adaptive strategies to evade acid-mediated toxicity, create an acidic environment that reduces the fitness of other normal and tumor populations (Fig. 3). Furthermore, we have previously demonstrated that regional acidosis can promote invasion through breakdown of extracellular matrix and can inhibit immune response to tumor antigens.

## Acidosis in carcinogenesis and cancer prevention

Here, we focus largely on Warburg physiology and its resulting acidosis in clinical cancers and their therapy. Less well-investigated are the evolutionary dynamics that select for aerobic glycolysis during carcinogenesis. Transition from normal tissue to invasive cancer is a multi-step process in which increasingly malignant cellular populations emerge over time generally coincident with accumulating genomic mutations. This is often described as “somatic evolution” because it appears formally analogous to Darwinian evolution in nature [19–22]. While this conceptual model is well accepted, the interactions with phenotypic properties and environmental selection forces that determine individual fitness remain ill-defined [18]. Both observational and computational analyses of intraductal carcinogenesis have found evidence for both hypoxia and acidosis. This is largely due to regional environmental variations in intraductal tumor caused by separation from blood vessels which remain in the ductal stroma separated from the tumor cells by the intact basement membrane [3, 23–25]. This is supported by expression of the hypoxia-induced glucose transporter, GLUT-1, and carbonic anhydrase isoform 9, CA-IX, adjacent to necrotic zones in DCIS as well as the upregulation of GLUT-1 and sodium hydrogen exchanger (NHE-1, a marker for extracellular acidosis) in regions of microinvasion [26]. Notably, adaptation to hypoxia and acidosis has been shown to be critical for the transition from *in situ* to invasive tumor in human cervical cancer [4].

Computer simulations [25] have demonstrated that changes in microenvironmental pH can slow the rate of evolution in *in situ* cancers. This was supported by a recent study showing that sodium bicarbonate added to drinking water in TRAMP mice dramatically delayed the transition from *in situ* to invasive prostate cancer [5].

## Role of tumor acidity in drug resistance

Microenvironmental acidity plays an important role in the response of malignant tumors to a wide variety of drugs and is likely a leading cause of chemotherapeutic failure in cancer treatment. A key factor in this resistance is the “reversed pH” gradient. That is, cancer cells are characterized by both an acidic extracellular pH (pHe) and a normal or alkaline cytoplasmic pH (pHi) [27, 28]. The alkaline pHi appears to confer resistance to both the hostile acidic milieu and drug cytotoxicity [29–33]. A number of studies have demonstrated that resistance to cisplatin and doxorubicin is associated with an elevation of pHi in multiple tumor cell lines (human epidermoid cancer, human prostate cancer, human ovarian cancer, and myeloma, a series of human lung and breast cancer cell lines) [33–37]. Similarly, cancer cell lines that are evolved to become drug resistant have a more alkaline pHi and a more acidic pH in subcellular organs when compared to the wild-type drug sensitive cells (HL60, K562, CEM, and MCF7) [38]. Many human spontaneous tumors have similar reversed gradients suggesting a clinical relevance for these studies [39]. While there are many potential mechanisms of resistance, it is clear that reversed pHe/pHi gradient interferes with the passage of drugs across the lipid bilayer of cells. Many anticancer drugs (such as doxorubicin and mitoxantrone) are weak bases which are neutralized and inactivated by protonation in the acidic microenvironment surrounding the cells or sequestered in intracellular acidic vesicles or endosomes [40–42]. An additional pH-dependent mechanism of drug resistance, recently described for cisplatin, includes both extracellular sequestration and exosomes mediated elimination of the drug from melanoma cells [43]. Interestingly, other studies have shown that an acidic pH increases the tumor cell exosomes release as well [44].

### Strategies of tumor cells to survive in an acidic environment

As noted above, cancer cells may use acid as a form of niche engineering in which they actively build an environment that is favorable for their own growth and survival but toxic to competitors and potential predators (such as the immune system). This appears to represent an evolutionary strategy termed “spite” in which an individual evolves a strategy that decreases its own fitness but with the benefit (in this case an acidic environment) that reduces the fitness of other normal and tumor populations and, thus, promotes growth and invasion. A key component of this putative evolutionary sequence is acquisition of adaptive strategies to evade acid-mediated toxicity [45]. These strategies include a series of proton export mechanisms, which are found both in the lipid bilayer of the external cell membrane and in intracellular compartmental membranes, including vacuolar type ATPase (V-ATPase) and the proton transporters NHE-1, monocarboxylate transporters (MCTs), CAs (mainly CA-IX), adenosine triphosphate synthase, Na(+)/HCO_3_(−) co-transporter, and the Cl(−)/HCO_3_(−) exchanger. These proton pumps are known to be overexpressed and/or overactivated in cancer cells when compared with their non-transformed counterparts. The availability of several inhibitors specific for these proton extrusion mechanisms has allowed investigation of their role in the maintenance of the reversed proton gradient and consequently in the acquisition of the malignant phenotype.

V-ATPase is an enzyme composed of multiple subunits, ubiquitously present in the membranes of vacuolar systems of animal cells. It is critical in vacuole acidification, thus, playing a crucial role in receptor-mediated endocytosis, intracellular trafficking of late endosomes, the transport of lysosomal enzymes from the Golgi apparatus to lysosomes, and the creation of the microenvironment necessary for proper protein transport, exchange, and secretion [46, 47]. V-ATPases can also be expressed in the plasma membrane of cancer cells [48–59] probably due to their enhanced exocytotic events and membrane-recycling mechanisms. Messenger RNAs and/or protein expression levels of different V-ATPase subunits have been shown to be increased in several cancer tissues and cell lines (human hepatocellular carcinoma, breast tumors and melanomas, esophageal squamous cancer cells, oral squamous cell carcinoma, human pancreatic carcinoma, and non-small cell lung cancer) compared with normal tissues [48, 51, 55, 60–64]. Moreover, the intensity of V-ATPase expression has been reported to associate to the pathological type and grade, both in non-small cell lung cancer and in pancreatic carcinoma [48, 55]. V-ATPase overexpression and its localization to the plasma membrane have been associated with the malignant phenotype in terms of invasiveness and metastatic potential and drug resistance [35, 48–50, 61, 62]. Recently, the increased expression of subunit of V-ATPases on the membrane of human melanoma cells deriving from metastatic lesions has been clearly shown [65] suggesting a role in cancer progression and in the metastatic cascade. These data may provide a new marker of tumor malignancy.

The membrane-bound NHEs represent another class of proteins that can extrude protons in exchange for a cation to maintain intracellular electroneutrality. They are present at the surface of most cells where they have a central role in regulating cellular volume and pH homeostasis. NHE isoform 1 (NHE-1) is the most common isoform of the NHEs family, and it is ubiquitous in all mammalian cells. In normal cells, NHE-1 activity is allosterically increased with decreasing pHi, resulting in rapid activation and subsequent elevation of pHi as a consequence of increased proton extrusion [66]. An aberrantly elevated NHE-1 activity has been correlated in tumors with pHe/pHi gradient reversal and in turn, associated with tumor origin, local growth, and further progression of the metastatic process [67, 68]. Molecular mechanisms underlying this tumor associated NHE-1 constitutive activation are only recently becoming evident. NHE-1 regulation occurs through the phosphorylation of key amino acids in the cytosolic domain as well as by its interaction with other intracellular proteins and lipids. Ultimately, NHE-1 regulators alter transport activity by altering its affinity for intracellular H^+^ such that it is more active at a more alkaline pHi [69]. In breast cancer cells, NHE-1 is highly expressed in invadopodia, invasive protrusions capable of proteolytic degradation of the extracellular matrix, where they play an essential role in creating the acidic extracellular microenvironment that facilitates proteases activity [70, 71]. As yet, large clinical studies examining NHE-1 expression in human tumors are lacking. However, recently NHE genes expression was found to be strongly upregulated in several lung cancer histotypes [60]. Interestingly, the expression change patterns have been reported to be highly complementary between NHE genes and the V-ATPase genes in different cancer types, suggesting that the NHE antiporters may play a complementary role to that of the V-ATPases [60].

Monocarboxylate transporters (MCTs) are proton symporters that transport monocarboxylates such as l-lactate, pyruvate, and the ketone bodies across the plasma membrane. There are four isoforms, MCTs 1–4, which are known to perform this function in mammals, each with distinct substrate and inhibitor affinities. MCTs play essential metabolic roles in most tissues, with their distinct properties, expression profile, and subcellular localization matching the particular metabolic needs of a tissue. They also play a key role in maintaining the pH homeostasis [72]. MCT1, MCT2, and MCT4 genes have been shown to be upregulated in several cancer histotypes (breast, colon, lung, ovary) with a considerable variation in the MCT isoforms expressed in different tumors [73, 74]. MCT1, MCT4, and their chaperone CD147 are overexpressed in the plasma membrane of glioblastomas compared with diffuse astrocytomas and non-neoplastic brain [75]. MCT1 and MCT4 both have elevated activity in human melanoma cells in response to low extracellular pH [76]. MCT1 has been reported to be upregulated in neuroblastoma cells, and elevated MCT1 mRNA levels have been detected in fresh neuroblastoma biopsy samples, with a positive correlation between expression level and risk of fatal outcome [77]. Xu et al. [60] recently reported MCT genes to be upregulated in breast, colon, liver, and two lung (adenocarcinoma, squamous cell carcinoma) cancers, but not in prostate cancer. Interestingly, lactate released as a waste product of glycolytic energy production in hypoxic tumor microenvironment has been demonstrated to constitute a prominent substrate that fuels the oxidative metabolism of tumor cells in oxygenated regions, and MCT1 has been shown to be involved in lactate uptake by a human cervix squamous carcinoma cell line that preferentially utilized lactate for oxidative metabolism [78].

Carbonic anhydrases (CA) and HCO_3−_ transporters have also been found to play a role in neutralizing the protons in cancer cells. The membrane-bound CAs catalyze the otherwise slow reaction from CO_2_+ H_2_O to H_2_CO_3_, which dissociates into HCO_3_^−^ (bicarbonate) and H^+^ in an acidic extracellular environment. The HCO_3_^−^ is then transported across the membrane through an HCO_3_^−^ transporter into the intracellular environment, where it reacts with a H^+^ to form CO_2_ and H_2_O; the CO_2_ is freely membrane-permeable and diffuses out of the cell, forming a cycle for removing excess H^+^ [79, 80]. CA isoform 9 is known to be inducible by hypoxia [81] and, unlike most other CA isoforms, is associated with many tumors [82, 83]. Very few normal tissues, with the exception of stomach [84], express significant levels of CA9 so that positive staining for CA9 is considered an established marker of tumor hypoxia and a clinical indicator of aggressive cancers (for example, breast and bone) with poor prognosis [85–87]. In addition to CA9, CA12 and CA14 genes have been recently reported to show upregulation in breast, colon, liver, and two lung (adenocarcinoma, squamous cell carcinoma) cancers (but not in prostate cancer), with two HCO_3_^−^ transporters, NBC2 (SLC4A5) and NBC3 (SLC4A7), also being upregulated in colon, liver, and two lung cancers types analyzed [60].

### Summary

Normal function of mammalian cells requires robust mechanism to regulate their pHi [88], by sensing changes and then rapidly responding by moving acids and/or bases across the plasma membrane. Cytosolic pH is extraordinarily important, affecting the ionization state of all intracellular weak acids and weak bases, a large number of cellular macromolecules including all proteins. Small perturbation in intracellular pH may potentially affect a wide array of biological processes. In the pathological process of cancer development, the acidification of tumor microenvironment represents an evolutionary advantage both for invasion and proliferation and for response to many chemotherapeutic treatments. Thus, upregulation of proton pumps is necessary to generate the slightly alkaline pHi and markedly acidic pHe, which are essential for cancer biology and response to treatment. As our understanding of these mechanisms increases so do opportunities for new cancer specific therapeutic targets.

## Proton exchangers as a therapeutic target

The reversal of pH gradient in cancer cells is increasingly considered as a hallmark of virtually all cancers [89–91]. And, thus, proton extrusion mechanisms represent appealing targets for new and less toxic anticancer treatment strategies [92]. Indeed, several studies have shown that targeting membrane proton pumps can cause cancer cell death, inhibit proliferation, reduce invasiveness and metastasis formation, and restore sensitivity of drug-resistant cancer cells to chemotherapeutics.

### V-ATPases inhibitors

Many studies have shown a key role of V-ATPases in drug resistance, cancer cells invasiveness, and in their capacity to migrate. Thus, there is much interest in the potential role of anti V-ATPases inhibitors as anticancer drugs, both as monotherapy and in combination with different chemotherapeutics [93, 94]. A growing number of V-ATPases inhibitors are reported to be effective against several cancer hystotypes. The first V-ATPase inhibitors to be discovered were bafilomycin and concanamycin [95]. However, these drugs and their subsequent derivatives have proven too toxic to be used as antitumor drugs. More recently, other V-ATPases inhibitors belonging to the benzolactone enamide class, such as salicylihalamide, lobatamides, and oximidines, have been described. With the achievement of total syntheses of salicylihalamide, lobatamide, and related compounds, the elaboration of congeners with specificity for particular enzyme isoforms may provide drug candidates that are less toxic [96, 97]. Limited supplies have so far precluded extensive *in vivo* testing of the benzolactone enamides.

An alternative approach for inhibiting the V-ATPase is silencing the expression of selected subunits using small interfering RNAs (siRNA) [54, 57, 98–100]. Isoform specific siRNAs employed to selectively target mRNAs isoforms preferentially expressed on cancer cells [54] might result in a less toxic cancer therapy.

### Proton pump inhibitors

**Fig. 4:**
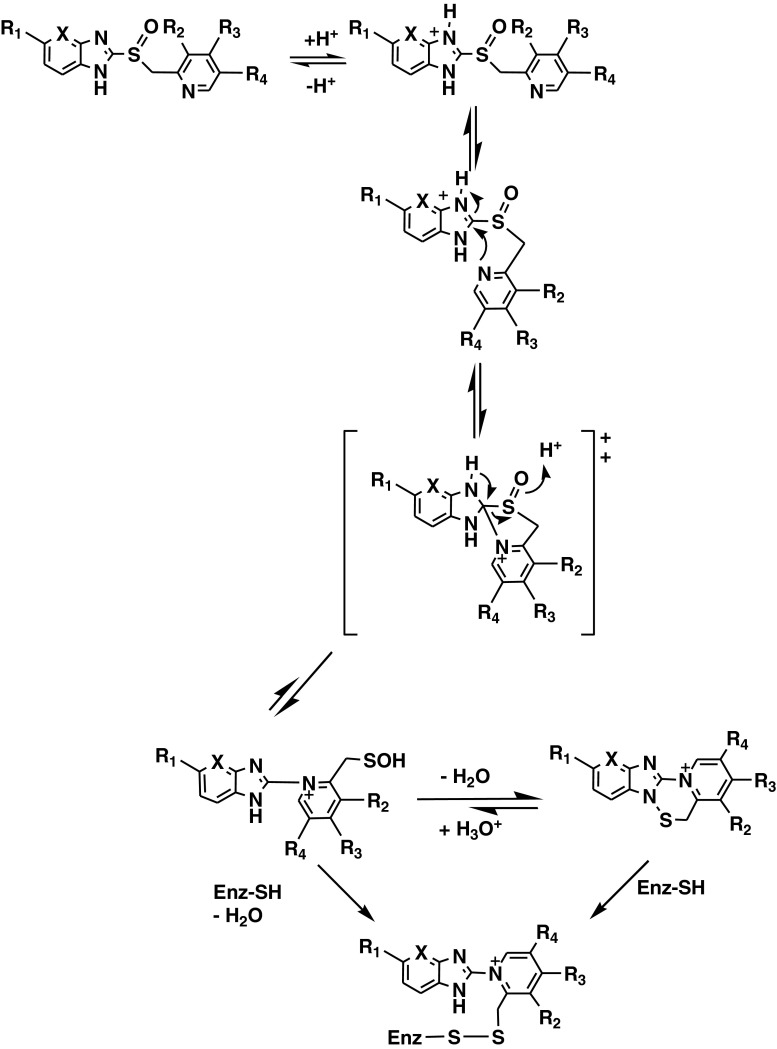
Proton pump inhibitors are activated by protonation in acidic microenvironment

Among several anti-V-ATPases approaches, the most promising results have been obtained with PPIs, a class of potent antiacidic drugs (Table 1), designed for treatment of peptic diseases. These drugs have been used by billions of people worldwide in the last decades, without significant side effects, even at high dosages (as in patients with Zollinger-Hellison syndrome). Interestingly, the absence of toxicity for this class of drugs is largely due to their dependence on an acidic pH for activation [101]. Thus, unlike the vast majority of the drugs including anticancer drugs, PPIs require an acidic environment for activation. As lipophilic and weakly basic prodrugs, they easily penetrate cell membranes and concentrate in acidic compartments, where they are unstable and are converted into sulfonamide forms which are the active inhibitors [102] (Fig. 4).

**Table 1:**
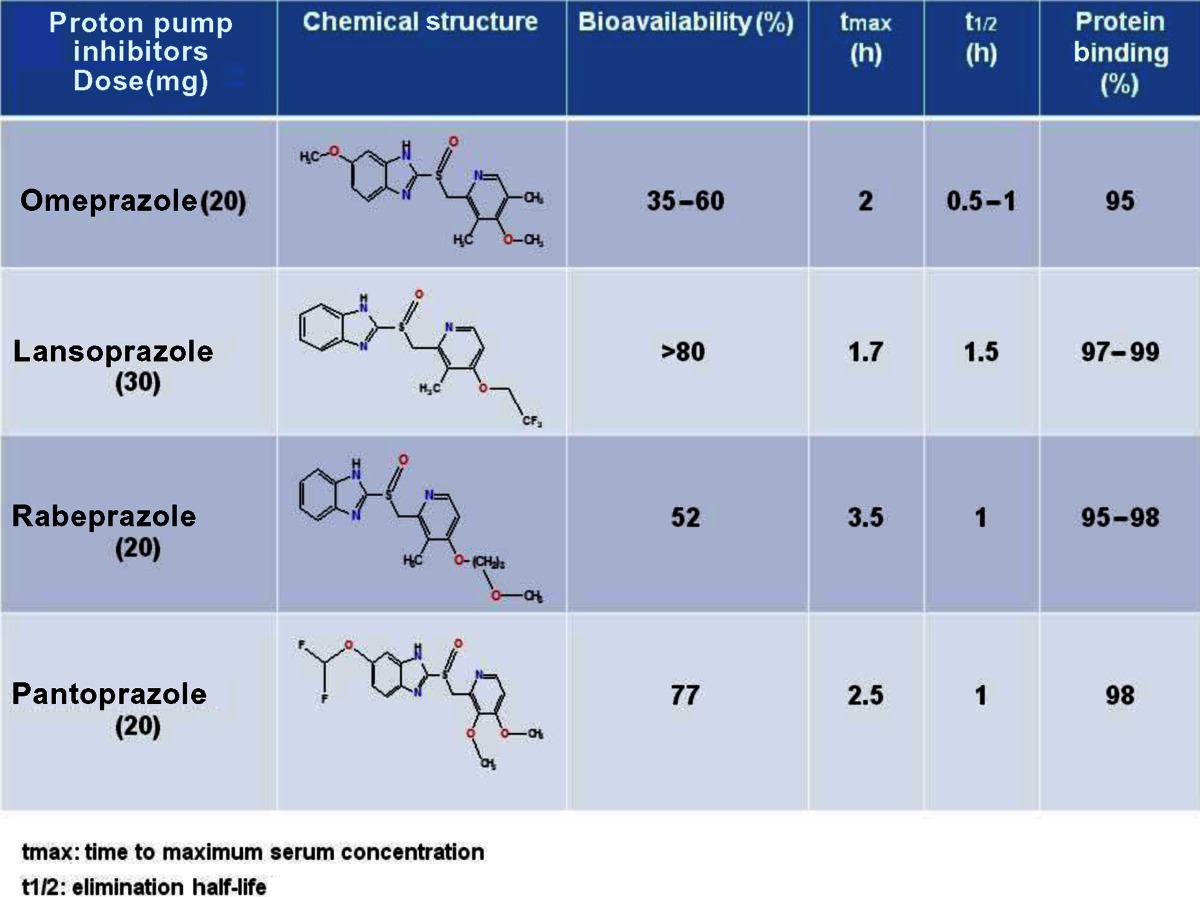
Comparison of pharmacokinetics of proton pump inhibitors

Based on these properties, PPIs have been extensively investigated for their potential to reduce tumor acidity and overcome the acid related chemoresistance. Furthermore, PPIs could have direct tumor cell toxicity by depriving them of a key mechanism for maintaining pHi/pHe gradient. A number of studies have now shown that PPIs can be useful in modulating tumor acidification and restoring chemotherapeutic sensitivity in drug-resistant cancer cells both *in vitro* and *in vivo* [58, 103–106]. These preclinical data have been supported by clinical studies in companion animals with spontaneous tumors [107] and in patients with osteosarcoma [103].

In addition, specific cytotoxic effects of PPIs on tumor cells have been reported [52, 65, 106, 108–112]. As expected, the PPI-induced cytotoxicity is strongly enhanced in low pH culture conditions [65]. PPIs’ activity has been investigated in several human tumor histotypes, such as melanoma [58, 65, 109], B cell lymphomas [108], pancreatic cancer [106], gastric carcinoma [104, 105, 110, 111], Ewing sarcoma [52], osteosarcoma [103, 112], rhabdomyosarcoma, and chondrosarcoma [112]. PPIs have also been shown to overcome the acidity-induced tumor immune escape mechanisms [113–115]. Finally, based on meta-analysis of observational studies and multicenter prospective cohort study, administration of PPIs in patients with Barrett’s esophagus significantly reduces the risk of esophageal adenocarcinoma and/or high grade dysplasia [116, 117].

### NHE-1 inhibitors

Inhibition of NHE-1 represents an additional potential target in anticancer therapy. Indeed, NHE-1 inhibitors have demonstrated efficacy in malignant glioma [118], hepatocellular carcinoma cells [119], and breast cancer cells [120]. Moreover, NHE-1 inhibition has been found to augment paclitaxel [91], imatinib [121], doxorubicin [122] and cisplatin [123] sensitivity in cancer cells. Amiloride was the first NHE inhibitor developed and has been shown to have a direct antitumoral and antimetastatic effect, *in vitro* and *in vivo* [124, 125]. Amiloride is a potassium-sparing diuretic, first approved in 1967 for management of hypertension and congestive heart failure. In the subsequent 40 years, it has been shown to be well tolerated and safe in humans. Cariporide is more recent but well studied specific and powerful NHE-1 inhibitor, for which an antitumor effect has been reported [68]. It has been shown to be useful in overcoming drug resistance and inhibiting the metastatic process [126]. Cariporide has undergone clinical trials in a cardiological setting and for ischemic reperfusion injury and is generally well tolerated. However, some side effects mainly related to drug accumulation and cerebrovascular complications have been reported. Importantly, the potency of cariporide and some other NHE-1 inhibitors are related to the ionization state of the guanidine residues. Thus, the acidic extracellular pH of tumors would be expected to augment the efficacy of these drugs, a potential advantage in terms of dose dependent side effects.

### Carbonic anhydrases inhibitors

CA9 is an attractive target for anticancer therapy, because it is selectively expressed by tumor cells and shows highly restricted expression in normal tissue. Pharmacologic interference of CA9 catalytic activity using monoclonal antibodies or CA9 specific small molecule inhibitors has been shown recently to impair primary tumor growth and metastasis. Among several classes of small molecules known to effectively inhibit CAs, compounds based on sulfonamide/sulfamates and coumarins, particularly chemotypes of these compounds selective for extracellular CAs such as CA9 [127, 128], have demonstrated a promise as potential anticancer agents. Treatment of hypoxic, metastatic 4 T1 mouse breast tumors with a fluorescent sulfonamide CA9 inhibitor resulted in a significant inhibition of tumor growth [129]. Furthermore, ureido sulfonamide [127] and glycosyl coumarin [128] inhibitors of CA9 produced significant inhibition of primary tumor growth in human and mouse models of orthotopic breast cancer. Treatment of HT-29 xenografts with the high affinity inhibitor of CA9 indanesulfonamide reduced tumor growth, and further regression was observed when the inhibitor was used in combination with radiotherapy [130]. Recent data suggest that sulfonamide and coumarin inhibitors of CA9 activity are also efficacious in reducing metastatic burden in preclinical models. For example, ureido sulfonamides significantly decrease lung metastases from breast cancer [127, 129], and similar results were achieved using glycosyl coumarins [128, 129].

### MCTs inhibitors

A number of inhibitors of MCTs have been described such as α-cyano-4-hydroxycinnamate (CHC) and its analogues, stilbene disulfonates including 4,4′-di-isothiocyanostilbene-2,2′-disulfonate (DIDS) and 4,4′-dibenzamidostilbene-2,2′-disulfonate (DBDS), phloretin, and bioflavanoids such as quercetin [72, 131]. However, none of these is specific for MCTs. CHC is a potent inhibitor of the mitochondrial pyruvate transporter [72], while DIDS and DBDS inhibit the chloride/bicarbonate exchanger AE1 much more powerfully than MCT1 [72, 131]. MCTs inhibition with CHC has resulted in a decrease of tumor cell pHi in *in vitro* melanoma and neuroblastoma models [76, 77]. Interestingly, CHC activity increased when cultivating cells in an acidic medium [76]. Inhibiting MCT1 with CHC or siRNA induced a switch from lactate fueled respiration to glycolysis in a human cervix squamous carcinoma cell line that preferentially utilized lactate for oxidative metabolism [78]. This last effect, together with a retarded tumor growth, was also observed *in vivo* inhibiting MCT1 in a mouse model of lung carcinoma and xenotransplanted human colorectal adenocarcinoma cells [78]. CHC has been reported to decrease glycolytic metabolism, migration, and invasion and to induce cell death in an *in vitro* glioblastoma model. A synergistic effect when combining CHC with temozolomide has also been reported in this model. The effectiveness of CHC in glioma cells appeared to be dependent on MCT membrane expression [75, 132]. Orthotopic application of the same inhibitor in immunodeficient rats after intracranial implantation of glioma cells has been shown to impair glioma invasion and to induce tumor necrosis and increase animal survival in an *in vivo* model.

## Tumor acidity as a therapeutic target

In addition to proton extrusion mechanisms, cancer microenvironmental can be viewed as a potential target for anticancer therapy and a number of potential strategies are available.

### Acridine orange

AO is known to accumulate densely in intracellular vesicles, especially lysosomes, in an acidity dependent manner. AO shows marked and prolonged accumulation in cancer cells, since these contain many strongly acidic lysosomes [133] and is, therefore, useful for visualizing tumor cells during surgery through a fluorescent microscope. Furthermore, it is used for photodynamic therapy, as it has a strong cytocidal effect on tumor cells following excitation through blue light or low dose radiation [134]. Kusazaki et al. [134–136] have developed an innovative approach using minimally invasive surgery combined with photo and radiodynamic therapy with AO for treatment of musculoskeletal sarcomas. They report improved postoperative limb function when compared with conventional surgery with wide tumor resection. Clinical pilot studies have yielded excellent results, with low local recurrence rates, good prognosis, and excellent limb function [136–139].

pH-Sensitive nanosystems for drug delivery in cancer therapy have been reviewed recently [140]. A variety of nanomaterials responding to physical, chemical, or biological stimuli have been synthesized and investigated as drug delivery systems (DDSs). Among these, pH-sensitive systems have been most widely used for drug delivery in cancer therapy. According to their constituents, nanomaterials can be classified as organic, inorganic, or hybrid. One approach optimizes intratumoral drug release using nanomaterials with “ionizable” chemical groups, such as amines, phosphoric acids, and carboxylic acids that undergo pH-dependent changes in physical or chemical properties resulting in drug release. An alternative approach uses acid-labile chemical bonds to covalently attach drug molecules directly onto the surfaces of existing nanocarriers or to construct new nanocarriers. These acid-labile chemical bonds are stable at neutral pH but are degraded or hydrolyzed in acidic media. Finally, a novel pH responsive DDSs incorporates carbon dioxide generating precursors that produce CO_2_ gas in an acidic environment, leading to disintegration of the carrier and release of drug molecules. This strategy is based on the fact that HCO_3_^−^ reacts with acid to produce carbonic acid, which easily decomposes to yield carbon dioxide (CO_2_) gas and water. Common CO_2_ generating agents include sodium bicarbonate and ammonium bicarbonate, both of which are compatible with normal cellular systems and the tumor microenvironment. Several studies have demonstrated that novel pH-sensitive drug delivery systems are capable of improving the efficiency of cancer treatment. A number of these have been translated from bench to clinical application and have been approved by the Food and Drug Administration for cancer treatment [140].

## Conclusion

Increased acid production is a consequence of increased anaerobic glucose metabolism in tumors that results from regional hypoxia due to disordered vascular development and the Warburg effect. The evolution of the latter during carcinogenesis is likely favored by the benefits of increased acid production which promotes invasion and proliferation of the cancer cells at the expense of their competitors and blunts the immune response. This “metabolic dysregulation” is now viewed as a “hallmark” [141] of cancer and confers an additional benefit of promoting tumor cell adaptation to many chemotherapies. However, this reversed acid gradient in cancers also provides an inviting target for new therapeutic strategies that are being examined in multiple centers throughout the world.

## Abbreviations

GLUT-1: Glucose transporter-1
CA: Carbonic anhydrase
NHE: Sodium hydrogen exchanger
pHe: Extracellular pH
pHi: Cytoplasmic pH
V-ATPase: Vacuolar-type ATPase
MCT: Monocarboxylate transporter
PPIs: Proton pump inhibitors
CHC: α-cyano-4-hydroxycinnamate
DIDS: 4,4′-di-isothiocyanostilbene-2,2′-disulfonate
DBDS: 4,4′-dibenzamidostilbene-2,2′-disulfonate
AO: Acridine orange
DDSs: Drug delivery systems
